# Optimization of the Extraction Conditions and Biological Evaluation of *Dendropanax morbifera* H. Lev as an Anti-Hyperuricemic Source

**DOI:** 10.3390/molecules23123313

**Published:** 2018-12-14

**Authors:** Seung-Sik Cho, Seung-Hui Song, Chul-Yung Choi, Kyung Mok Park, Jung-Hyun Shim, Dae-Hun Park

**Affiliations:** 1 Department of Pharmacy, College of Pharmacy, Mokpo National University, Muan, Jeonnam 58554, Korea; sscho@mokpo.ac.kr (S.-S.C.); tmdgml7898@naver.com (S.-H.S.); 2 Jeonnam Institute of Natural Resources Research, Jangheung-gun, Jeonnam 57922, Korea; blockstar@hanmail.net; 3 Department of Parmaceutical Engineering, Dongshin University, Naju, Jeonnam 58245, Korea; parkkm@dsu.ac.kr; 4 Department of Nursing, Dongshin University, Naju, Jeonnam 58245, Korea

**Keywords:** *Dendropanax morbifera* leaf, xanthine oxidase, hyperuricemia, HPLC

## Abstract

*Dendropanax morbifera* H. Levis a medicinal plant native to South Korea, East Asia, and South America. Among some 75 species, one species grows in Korea. In previous studies, *D. morbifera* extracts with anti-oxidant, anti-inflammatory, anti-complementary and anti-cancer activities were reported. The present study aims to investigate optimization of extraction and evaluation of anti-hyperuricemic effects of *D. morbifera* leaf and the phytochemicals contained therein. Ethanol and hexane extract were found to display the best xanthine oxidase inhibition among six types of solvent and water extract. The antioxidant effect of the ethanol extract was superior to that of the hexane extract. The DPPH radical scavenging effect of the ethanol and hexane extracts were 81.52 ± 1.57% and 2.69 ± 0.16. The reducing power of the ethanol and hexane extracts were 9.71 ± 0.15 and 0.89 ± 0.01 mg/g equivalent of gallic acid. Total phenols of the ethanol and hexane extracts were 6.53 ± 0.16 and 0.63 ± 0.001 mg/g equivalent of gallic acid. In addition, we compared the two marker compounds from *D. morbifera*, chlorogenic acid and rutin, which were determined in the ethanol extract at 0.80 ± 0.03% and 0.52 ± 0.01%, respectively. We found that the ethanol extracts showed better xanthine oxidase inhibition than hexane extracts. Especially, ethanol extracts showed higher antioxidant activity than hexane extracts. Based on these results, we selected the ethanol extract as an effective xanthine oxidase inhibitor and confirmed whether ethanol extracts showed xanthine oxidase inhibition in animal experiments. The in vivo mouse study demonstrated that ethanol extract of *D. morbifera* leaf at the dose of 300 mg/kg could inhibit blood/hepatic xanthine oxidase activity and this result shows that the xanthine oxidase inhibitory activity in vitro is reproduced in vivo. The present study showed that ethanol extract was optimal xanthine oxidase inhibitor which can be applied to prevent diseases related to hyperuricemia.

## 1. Introduction

*Dendropanax morbifera* (Aralicaceae), called ginseng tree, is a perennial tree that grows in forests in the southern regions in South Korea. The leaf, stem, and root of *D. morbifera* have been used in traditional medicine to treat infectious diseases, dermatopathy, dysmenorrhea and migraine [1,2]. To date, a few studies have been carried out investigating *D. morbifera* as a functional food and medicinal source [2]. This plant has been reported to exhibit various pharmacologic effects including antioxidant, anticancer, anti-inflammatory, anticomplementary, anti-amnesic, and antidiabetic activities [3,4,5,6]. However, systematic evaluation of pharmacological efficacy based on active constituents and the standardization of *D. morbifera* remains insufficient.

Recently, we found that the extract of *D. morbifera* leaf exhibited in vitro xanthine oxidase inhibitory activity, indicating that it could serve as a functional source for anti-hyperuricemia agents [2]. Hyperuricemia involves excessive uric acid levels in the blood, which are due to the abnormal intake of food with high purine content, and these excessive uric acid levels are consistently accompanied by gout and metabolic syndrome [7]. Uric acid is formed by the oxidation of hypoxanthine to xanthine and of xanthine to uric acid by xanthine oxidase (XO) [8]. High levels of uric acid by XO lead to hyperuricemia, which is a main cause of gout. Gout is a metabolic disorder that is strongly associated with high levels of uric acid in the body, and it can cause diabetic cardiomyopathy, arthritis, and nephrolithiasis [9].

In our recent study, the optimized extraction and analysis method of marker compounds in *D. morbifera* leaf was established [2]. Additionally, we identified the common components of *D. morbifera* leaf from four Korean production sites and compared the extraction yield and effective marker content by region [2]. In the present study, we investigated the optimum extraction of *D. morbifera* leaf as well as the biological activities of extracts from *D. morbifera* leaf to evaluate their feasibility as an anti-hyperuricemic source. The optimized extract from *D. morbifera* leaf was prepared and evaluated for its antioxidant and XO inhibitory activities of *D. morbifera* leaf extract in vivo.

## 2. Results and Discussion

### 2.1. In Vitro Xanthine Oxidase Inhibitory Activities of D. morbifera Extracts

The effects of the various solvent extracts on the XO inhibitory activity of *D. morbifera* are shown in Table 1. Allopurinol (ALP, positive control) at a concentration of 50 μg/mL significantly decreased the uric acid concentration (4.04 ± 1.49%). The XO inhibitory activities of the hexane and ethanol extracts were significantly higher than those of the other extracts at the concentration of 2 mg/mL (38.7 and 37.3% inhibition). According to previous reports, in the case of XO inhibition at 1 to 2 mg/mL, the plant extracts showed significant results in animal experiments [10]. We previously reported various plant sources as potential XO inhibitors [11]. Yoon et al. [10,12] reported that the extracts of *Corylopsis coreana* and *Camellia japonica* each inhibited XO activity by approximately 50% at a concentration of 2 mg/mL. Yoon et al. [11] also reported that *Quercus acuta* extract showed approximately 50% XO inhibitory activity at a concentration of 1 mg/mL, and that *Cudrania tricuspidata* extract inhibited XO by approximately 75% at a concentration of 2 mg/mL [13]. All of the above results showed in vitro and in vivo correlation of XO inhibition. However, further investigation on the clinical and biomedical relevance of our previous and present results is needed.

Therefore, we considered that the hexane and ethanol extracts would inhibit XO in animal models, and performed an antioxidant test to determine the optimal results in an animal model. The antioxidant activity of plant material is known to play important roles in hyperuricemia and gout. Thus, we compared the antioxidant capacities of the hexane and ethanol extracts and tested the best materials for an in vivo test.

### 2.2. Antioxidant Activity and Total Phenolic Contents of D. morbifera Extracts

According to previous reports, the antioxidant effects of plant extracts have curative benefits against conditions such as inflammation, oxidative stress, and other metabolic diseases such as hyperuricemia and gout arthritis [10,14,15]. Thus, we compared the antioxidant effects of the hexane and ethanol extracts. First, we compared the DPPH radical scavenging activities between the hexane and ethanol extracts. The ethanol extract showed an antioxidant activity 30 times higher than the hexane extract. Reducing power is one of the tools used to evaluate an antioxidant effect. The ethanol extract showed a reducing power 11 times higher than the hexane extract. Furthermore, phenolic-rich sources of phytochemicals with antioxidant activities have curative benefits against various metabolic diseases. In the present study, the ethanol extract showed total phenolic contents 10.3 times higher than the hexane extract (Table 2). Based on the antioxidant data, it was concluded that the ethanol extract had an antioxidant ability that was better by 10 times or more than the hexane extract. Considering that its xanthine oxidase inhibition activity is similar to that of the hexane extract, it is considered that the ethanol extract is excellent for the development of an anti-hyperuricemic material.

### 2.3. Contents of Marker Compounds in D. morbifera Leaf Extracts

In a previous report, we identified chlorogenic acid and rutin as marker compounds of the extracts of *D. morbifera* leaves [2]. This finding could be important in industrial uses of this plant. Based on a chromatographic measurement, we found that the two indicator substances were not detected in the hexane extract, but rutin (0.52%) and chlorogenic acid (0.8%) were detected in the ethanol extract. Rutin and chlorogenic acid are known to have diverse pharmacological effects, such as antihyperuricemic and anti-inflammatory activities [16,17,18,19]. In the present study, we compared the rutin and chlorogenic acid contents between the ethanol and hexane extracts; rutin and chlorogenic acid were both found in the ethanol extract (Table 3). In our previous study, we set chlorogenic acid and rutin as indicators of *D. morbifera*. Two markers were set as markers for identification and quality control of *D. morbifera* extract [2]. In the present study, the presence of two substances in hexane extract could not be confirmed, however xanthine oxidase inhibition had activity similar to ethanol extract. This result implies that there is another xanthine oxidase inhibitor in the hexane extract. We will use a bioassay guided purification method in hexane extract as a further study to find another marker compound.

### 2.4. In Vivo XO Inhibitory and Antihyperuricemic Effects of Ethano Extract from D. morbifera Leaf

**Figure 1:**
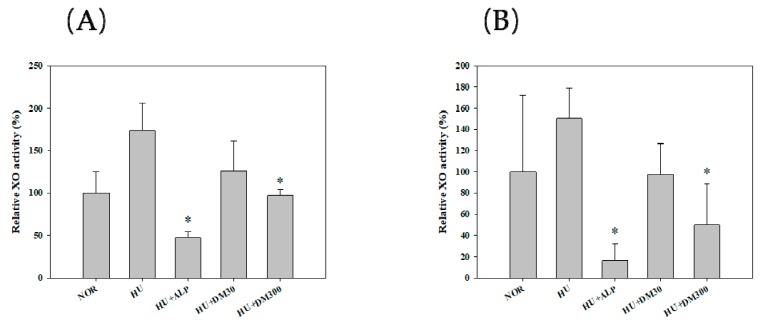
Relative activity of hepatic (**A**) and serum (**B**) xanthine oxidase (XO) after the oral administration of saline in normal mice (NOR) and after the oral administration of saline (HU), allopurinol at a dose of 10 mg/kg (HU + ALP), or DM at doses of 30 mg/kg (HU + DM30) and 300 mg/kg (HU + DM300) in hyperuricemic mice for 7 days. The rectangular bars and their error bars represent the means and standard deviations, respectively (*n* = 5). The asterisks indicate values that are significantly different from those of the HU group (*p* < 0.05).

Figure 1 shows the effects of the ethanol extract on the hepatic and serum XO activities in potassium oxonate-induced hyperuricemic mice. The one-week oral administration of allopurinol (by 47%) and ethanol extract at a dose of 300 mg/kg (by 97%, respectively) significantly reduced hepatic XO activity in comparison to the hyperuricema group (*p* < 0.05). Similarly, the one-week oral administration of allopurinol (by 16%) and ethanol extract at a dose of 300 mg/kg (by 50%, respectively) significantly reduced plasma XO activity in comparison to the hyperuricemia group (*p* < 0.05). However, ethanol extract at a dose of 30 mg/kg did not show statistical significance as compared to the hyperuricemia group. Thus, the 300 mg/kg treatment of ethanol extract was considered to show XO inhibitory activity. In an in vitro test, the ethanol extract showed a significant XO inhibitory effect and a consistent XO inhibitory effect in an in vivo test.

**Figure 2:**
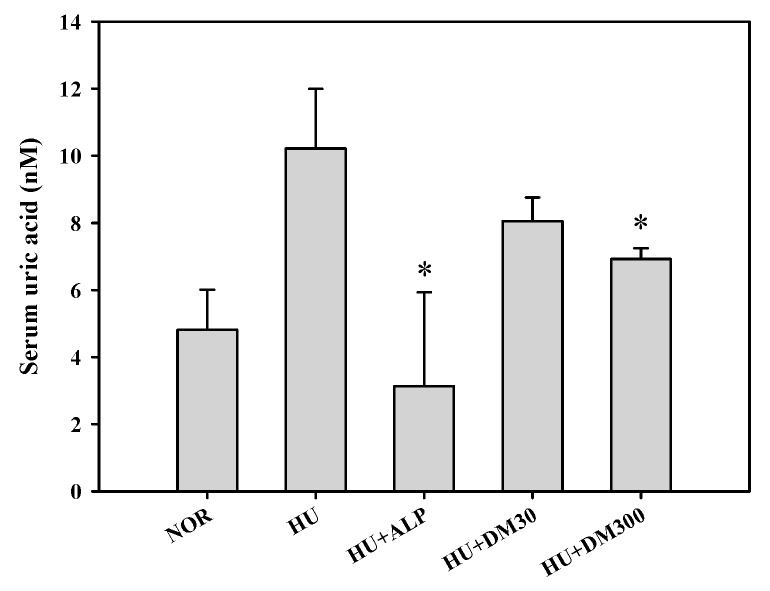
Serum uric acid levels after the oral administration of saline in normal mice (NOR) and after the oral administration of saline (HU), allopurinol at a dose of 10 mg/kg (HU + ALP), or DM at doses of 30 mg/kg (HU + DM30) and 300 mg/kg (HU + DM300) in hyperuricemic mice for 7 days. The rectangular bars and their error bars represent the means and standard deviations, respectively (*n* = 5). The asterisks indicate values that are significantly different from those of the HU group (*p* < 0.05).

Figure 2 shows the effects of the extract on the serum uric acid levels in the same animal model. Notable, the 300 mg/kg treatment of the extract exhibited a significant antihyperuricemic effect in an in vivo test.

We considered the oral intake of ethanol extract of 1.46 g daily to help in preventing hyperuricemia and gout. The oral dose for a human weighing 60 kg is 1460 mg/day (24.33 mg/kg/day). The conversion factor between humans and mice is known to be 12.33. Therefore, if the effective dose for mice is 300 mg/kg/day, the human equivalent dose is 1.46 g/60 kg/day of ethanol extract or 15.2 g/60 kg/day as dried leaf. Thus, we concluded that the oral intake of 1.46 g of the extract of *D. morbifera* leaves is beneficial for preventing and/or decreasing the possibility of the occurrence of hyperuricemia-related disease. Taken together, we found beneficial effects of the extract of *D. morbifera* leaves from the results of biological evaluation through the antioxidant assay and XO assay in vitro and in vivo. We optimized the basic extraction condition due to antioxidant and XO inhibitory activities and the contents of marker compounds as XO inhibitors (e.g., rutin and chlorogenic acid). In addition, we have applied optimized extracts to in vivo hyperuricemic mouse models and found that the XO in liver and plasma was inhibited. In previous reports, rutinwas shown to have the ability to draw out dose-dependent hypouricemic effects by exerting signifi­cant inhibitory effects on XO [20]. Meng et al. described that chlorogenic acid showed an anti-gout effect due to XO inhibition [18]. In addition, chlorogenic acid has shown an anti-inflammatory effect via the suppression of levels of proinflammatory cytokines such as interleukin (IL)-1β, IL-6, and tumor necrosis factor (TNF)-α induced by uric acid [18,19,21]. These results suggest that chlorogenic acid exerts dual effects in anti-gout and gout inflammatory diseases. Phenolic compounds are also known to be closely related to the prevention and treatment of diseases such as inflammation and gout. Phenolic contents are also associated with antioxidant and XO inhibition [10]. In the present study, we compared the antioxidant activity of hexane and ethanol extracts, and the ethanol extract showed higher phenolic content than the hexane extract. Therefore, it was thought that phenolic contents and antioxidant capacity were beneficial in XO inhibition, and our result was thought to confirm the results of animal experiments. Based on these results, it is necessary to study the function mechanism of XO and the single component from *D. morbifera* leaf that is inhibiting XO.

## 3. Experimental Section

### 3.1. Plant Materials

*D. morbifera* leaves were provided by the Jeollanamdo Wando provincial government in Jeonnam, Korea. A voucher specimen (MNUCSS-DM-02) was deposited in the Mokpo National University (Muan, Korea). The leaves were separated for the present study. The dried *D. morbifera* leaf (50 g) was extracted twice with each of hexane, ethyl acetate, acetone, methanol, ethanol, and hot water (250 mL, *v*/*v*) for 72 h. The yields of hexane, ethyl acetate, acetone, methanol, ethanol, and hot water extracts were 1.4, 1.8, 6, 9, 9.6 and 13% (*w*/*w*), respectively. Each resulting sample was filtered, the solvent was evaporated, and the water extract was freeze dried. For in vivo evaluation, *D. morbifera* leaf (200 g) was extracted twice with ethanol (2000 mL, *v*/*v*) at room temperature for 72 hours and evaporated in vacuo (50 °C). All samples were stored at 4 °C.

### 3.2. Animals

Male ICR mice (four-weeks old) were purchased from Samtaco Co. (Osan, Korea). The mice were kept in a clean room at a temperature of 20–23 °C with a relative humidity of 50 ± 5%. The mice were housed in ventilated mice cages (Tecniplast USA, Inc, West Chester, PA, USA) under filtered and pathogen-free air, with diet (Agribrands Purina Korea, Inc., Sungnam, Korea) and water available ad libitum. All animal experiments were carried out according to the Guidelines of the Animal Investigation Committee of Jeonnam Bioindustry Foundation (Naju, Korea) (approval number: JINR1503).

### 3.3. DPPH Free Radical Assay

The DPPH radical scavenging assay was evaluated to compare antioxidant activity of the extracts [10]. Briefly, sample solutions (0.5 mL) were mixed with 0.4 mM DPPH (0.5 mL) for 10 min. The absorbance at 517 nm was measured using a microplate reader (Perkin Elmer, Waltham, CT, USA). The radical scavenging activity was calculated in the form of a percentage using the following equation:

DPPH radical scavenging activity (%) = [1 − (A_sample_/A_blank_)] × 100 (1)

### 3.4. Reducing Power

The reducing power was used to evaluate the antioxidant activities of the extracts. Sample was mixed with 0.2 M sodium phosphate buffer and 1% potassium ferricyanide, followed by incubation at 50 °C for 20 min. Ten percent trichloroacetic acid solution was used and stop solution. Reaction mixture was centrifuged at 2000× *g* for 10 min, the supernatant was mixed with distilled water and 0.1% iron (III) chloride solution. Reaction mixture was measured at 700 nm. The reducing powers of the extracts were expressed as vitamin C equivalents [10].

### 3.5. Total Phenolic Content

The total phenolic content was determined using the Folin-Ciocalteu assay [10]. A sample and gallic acid (as standard) was mixed with 2% sodium carbonate and 10% Folin-Ciocalteu phenol reagent for 10 min. The absorbance of the mixture was then measured at 750 nm. The results were expressed as milligrams of gallic acid equivalents per gram of the sample 

### 3.6. Chromatographic Conditions

Analysis of samples were performed using high performance liquid chromatography (Alliance 2695 HPLC system, Waters, Milford, CT, USA). A Zorbax RP C18 analytical column (5 μm, 150 mm × 5 mm) was used with a mobile phase consisting of a mixture of acetonitrile and 0.2% phosphoric acid. A gradient elution (from 10/90 to 80/20, *v*/*v*) at a flow rate of 0.8 mL/min was used under the same analytical conditions previously described [2].

### 3.7. Determination of In Vitro Xanthine Oxidase (XO) Inhibitory Activity

The XO inhibitory assay was performed as follows: the assay system consisted of phosphate buffer (0.6 mL, 100 mM; pH 7.4), various concentrations of extract (0.1 mL), XO (0.1 mL, 0.2 U/mL), and xanthine (0.2 mL, 1 mM; dissolved in 0.1 N NaOH). Each mixture was shaken for 15min and a stop buffer (0.2 mL, 1 M HCl) was added at 290 nm. Allopurinol was used as a positive control [22].

### 3.8. Preparation of Hyperuricemia Model and Drug Administration

The mice were divided into five groups; NOR: normal, HU: hyperuricemic mouse, ALP: allopurinol (10 mg/kg) treatment group, DM30: DM (ethanol extract of *D. morbifera* leaf) 30 mg/kg treatment group, DM300: DM 300 mg/kg treatment group (*n* = 5 for each group). Hyperuricemia was induced via an intraperitoneal injection of potassium oxonate [23]. Briefly, the DM (30, 300 mg/kg) or allopurinol (10 mg/kg) were dissolved in 0.3% carboxymethylcellulose sodium solution. The allopurinol and DMs were orally administered once per day for seven days. Food (but not water) was withdrawn from the mice at 1.5 h prior to the drug administration, and mice were intraperitoneally injected with potassium oxonate (300 mg/kg) at 1 h before the last drug treatment on the seventh day in order to make hyperuricemic mice. Blood samples were collected at 1 h after the last drug treatment on the seventh day. The blood samples were allowed to clot for approximately 1 h at room temperature, then centrifuged at 10,000× *g* for 15 min in order to obtain serum. The serum samples were stored at −80 °C until use. Serum uric acid concentration was measured using standard diagnostic kits (Abcam, Cambridge, UK). Each assay was performed in triplicate.

### 3.9. Determination of In Vivo Xanthine Oxidase (XO) Inhibitory Activity

The residual activity of XO in the mouse liver and plasma were determined as reported previously [8]. Mice livers (0.5 g) were homogenized in a 1 mL aliquot of 50 mM sodium phosphate buffer (pH 7.4). The homogenates were centrifuged at 3000× *g* for 10 min at 4 °C. The supernatant was centrifuged at 10,000× *g* for 60 min at 4 °C, and was used for determining XO residual activity and total protein. An aliquot of xanthine solution (0.12 mL, 250 mM) was added to a test tube containing liver homogenate (10 μL) and potassium oxonate solution (0.54 mL, 1 mM) in sodium phosphate buffer (50 mM, pH 7.4) that had been previously incubated at 35 °C for 15 min. The reaction was stopped by adding a 0.1 mL of 0.6 M HCl. Thereafter, the mixture was centrifuged at 3000× *g* for 5 min and finally measured at 295 nm. The total protein concentration was determined through the Bradford method [24]. XO activity was expressed as micromoles of uric acid formed per minute (U) per milligram protein.

### 3.10. Statistical Analysis

A *p*-value less than 0.05 was considered to be statistically significant using a *t*-test between the two means for the unpaired data or an ANOVA (post hoc test: Tukey’s multiple range test) among the three or more means for the unpaired data. All data were expressed as mean ± standard deviation and rounded to two decimal places.

## 4. Conclusions

In the present study, various solvent extracts of *D. morbifera* leaf were prepared. We selected hexane and ethanol extracts as XO inhibiting candidates, and their biological activities, such as antioxidant effects, XO inhibition, and contents of maker compounds were evaluated. The ethanol extract exhibited the most potent DPPH radical scavenging activity, reducing power, phenolic content, and XO inhibitory activity. Marker compounds such as chlorogenic acid and rutin were found in the ethanol extract as well. We confirmed the inhibition of XO in animal models, and found significant results on liver and plasma. Further investigation is warranted to confirm the in vivo mechanism study of *D. morbifera* extract, identify another XO, and assess the safe use of this plant.

## Figures and Tables

**Table 1:** In vitro xanthine oxidase inhibitory activity of various solvent extract.

Extract	Relative Activity (%)
Control	100 ± 10
ALP	4.04 ± 1.49
Ethyl acetate	67.2 ± 1.2
Hexane	61.3 ± 1.9
Acetone	66.8 ± 0.9
MeOH	77.6 ± 1.0
Water	96.2 ± 4.1
EtOH	62.7 ± 3.9

**Table 2:** Antioxidant activities of hexane and ethanol extract from *D. morbifera* leaf.

	DPPH Acavenging (%)	Reducing Power (mg/g eq GA)	Total Phenol (mg/g GA)	Total Flavonoid (mg/g QT)
Hexane ex	2.69 ± 0.16	0.89 ± 0.01	0.63 ± 0.00	ND *
Ethanol ex	81.5 ± 1.6	9.71 ± 0.15	6.53 ± 0.16	ND *

* ND (not detected).

**Table 3:** Comparison of marker compounds of hexane and ethanol extract from *D. morbifera* leaf.

Extract	Chlorogenic Acid (%, *v*/*v*)	Rutin (%, *v*/*v*)
Hexane	ND *	ND *
Ethanol	0.80 ± 0.03	0.52 ± 0.01

* ND (not detected).
